# Radical Cure of Consumption

**Published:** 1860-09

**Authors:** 


RADICAL CURE OF CONSUMPTION.—Dr. Guirette, of Lyons, is at the present time engaged in a series of experiments, in the wards of M. Piorry, at the Hôpital de la Charité, Paris, the recent seat of the quackery of the "Black Doctor," in order to test the value of a new treatment for the radical cure of pulmonary phthisis during its suppurative stage. This method, writes the Paris correspondent of the *Lancet,* "consists in the establishment of a fistulous opening through the integuments of the thorax and the pleura into the lung at the diseased part, and in the free admission of atmospheric air into the cavity of the abscess, which at the same time discharges its contents externally. Dr. Guirette was led to believe in the feasibility of such a plan by a pure accident. Having, at the hospital at Lyons, applied an issue to the chest of a phthisical patient, over the site of a cavity, and having inserted a pea in order to keep up the counter-irritation, this practitioner found that the foreign body had worked its way into the lung, and caused the pus contained in the abscess, which was a very superficial one, to escape at the artificial opening. The result, so far from being fatal to the patient, was so beneficial as to lead to complete recovery. He left the hospital apparently cured, and emigrated to Rio Janeiro."

"Since the occurrence of this case, Dr. Guirette has applied the penetrating cautery in three instances, and each time with the best success. Encouraged by this experience, he has come to Paris to submit his plan to public criticism; and although he has met with many rebuffs, is now enabled, by the kindness of M. Piorry, to plead his own cause, or rather that of his new system of treatment of this most incurable complaint. Operations have already been commenced; and a lad, aged about eighteen years, with all the symptoms of an abscess under the left clavicle, has been handed over to Dr. Guirette, who applied, on the third of June, a cautery of Vienna paste in the third intercostal space, toward the axilla. This, when the eschar falls, will be replaced by a pea attached to a thread of silk."

So far as the experiments have gone they appear to hold out promise of good results. An English medical gentleman has written to M. Piorry, relating a case of spontaneous formation of fistules during the last stage of phthisis. The prognosis in this case is favorable, and adds some support to Dr. Guirette's views.

We shall watch with interest the reports of the progress of the case, and in a future number place the results of this plan of treatment before our readers.

